# Physical Activity and Exercise Accelerometers reveal peculiar patterns of Physical Activity in cognitively impaired vs. healthy older adults

**DOI:** 10.1093/geroni/igaf122.2731

**Published:** 2025-12-31

**Authors:** Massimiliano Pau, Valeria Putzu, Gesuina Asoni, Daniela Viale, Benedetta Brandas, Maria Chiara Fastame

**Affiliations:** University of Cagliari, Cagliari, Sardegna, Italy; ASL Cagliari, Cagliari, Sardegna, Italy; ASL Cagliari, Cagliari, Sardegna, Italy; ASL Cagliari, Cagliari, Sardegna, Italy; University of Cagliari, Cagliari, Sardegna, Italy; University of Cagliari, Cagliari, Sardegna, Italy

## Abstract

Although there is consensus regarding the association between Physical Activity (PA) and cognitive performance of older adults, quantitative data regarding amount and intensity of PA performed by those with cognitive impairments.under free-living conditions, are scarce. We investigated the effects of cognitive impairment on several PA metrics and analyzed the patterns of mobility in terms of hourly variation using accelerometers in 104 community-dwelling older adults aged 60-85 stratified into three groups: cognitively impaired living in urban area (CI, n = 27), cognitively intact living in urban area (HC, n = 33) and cognitively intact living in the Blue Zone of Sardinia (Italy), a cluster of rural communities characterized by exceptional longevity (BZ, n = 44). All participants were monitored for 7 consecutive days using a clinically validated wearable accelerometer. Raw data were processed to calculate percentage of time spent in sedentary behavior (SB), light PA (LPA) and moderate-to-vigorous PA (MVPA). The results showed that older adults of the CI groups spend 76% of the day in SB, a value significantly higher than both HC (67%) and BZ (66%), and only 3.4% in MVPA, which was significantly lower than what observed in the BZ group (12.1%). Differences were also found in the propensity to mobility during the day, as indicated by the different hourly PA patterns. Such findings suggest that CI is associated with a sedentary lifestyle. The large differences observed in MVPA with those living in the BZ, suggest that activities of sufficient intensity might contribute to mitigate the effects of cognitive deficit and contribute to longevity.

